# Authorship issues continued…

**DOI:** 10.4103/0970-0358.73481

**Published:** 2010

**Authors:** Anup Mohta

**Affiliations:** Department of Pediatric Surgery, Chacha Nehru Bal Chikitslaya and Maulana Azad Medical College, Delhi-110 031, India

Sir,

I read with interest the editorial, “The question of authorship: Whose research is it anyway?[1] The author has very clearly summarized the authorship criteria as recommended by the International Committee of Medical Journal Editors and World Association of Medical Editors.

Sharing the issues of an ex-resident publishing the date from previous Institute, I have also come across a former resident publishing a paper using data and photograph of a patient from an Institute where he had worked previously. In this instance, the author failed to give credit to original Institute. The issue came to notice when another paper using the same photograph was published by the original Department.

As regards the research done in a Department, I have a few queries:

1. A postgraduate resident pursues a thesis under the guidance of his/her guide. More often than not, the guide conceives the idea of research; dictates the protocol; supervises day-to-day progress; is responsible for progress and is responsible for finally written document. Who should be the first author when the work is finished and a paper is sent for publication? Another fall out due to this “first author” issue is evident when the guide appears for his promotional interview and research carried out on his/her concept under his/her guidance is not credited to the individual, but his resident by the experts. This dissuades the faculty to give “good research ideas” to the residents and save them for their personal research instead.
2. If the resident is not interested in writing a paper out of the research or leaves the Department and the guide decides to publish the work, is he bound to give authorship to the resident?
3. In another scenario, if a clinician decides to conduct a clinical study in the patients involving operative work and long-term follow-up, is he not obliged to give authorship rights to the residents who assist him and do the follow up for the clinician? An example of this situation may be a study[2] published in the same issue of *Indian Journal of Plastic Surgery*. The author needs to be complimented for conceiving the new procedure and an algorithm. However, I am intrigued by repeated use of “we”, “our”, “us” in a single author study on hypospadias with about 2 years of follow-up. Is it just the language?

I look forward to some answers to my observations!

## References

[1] Thatte M (2010). The question of authorship: Whose research is it anyway?. Indian J Plast Surg.

[2] Bhattacharya S (2010). Amodified tubularized incised plate urethroplasty technique and a revised hypospadias algorithm. Indian J Plast Surg.

